# Comparing Missing Data Strategies for Generalized Pairwise Comparisons in Randomized Clinical Trials: A Simulation Study

**DOI:** 10.1002/sim.70737

**Published:** 2026-09-17

**Authors:** Ruben P. A. van Eijk, Ying Lu

**Affiliations:** ^1^ Department of Biomedical Data Science and Centre for Innovative Study Designs, School of Medicine Stanford University Stanford California USA; ^2^ Department of Neurology, UMC Utrecht Brain Center University Medical Center Utrecht Utrecht the Netherlands

**Keywords:** amyotrophic lateral sclerosis, clinical trials, combined assessment of function and survival, generalized pairwise comparisons, hierarchical endpoint, win statistics

## Abstract

Generalized pairwise comparisons are increasingly being used in randomized clinical trials. This study evaluates how missing data strategies impact the operating characteristics of endpoints combining overall survival and a longitudinal outcome. A simulation study was conducted to evaluate the impact of censoring and missing longitudinal data on the Type I error, power, and bias of a hierarchical composite endpoint. Seven missing data strategies were identified from the literature, including assigning ties to missing patient pairs, comparing patients at their last common visit, and applying multiple imputation. Conditional longitudinal and survival data were generated based on the natural history of amyotrophic lateral sclerosis. Simulation scenarios varied in censoring rates, extent of missing longitudinal data, differential attrition between treatment arms, and the dependency of missingness on disease severity. All methods were unbiased and maintained nominal Type I error when censoring and missingness were balanced across treatment arms. Under imbalance, however, Type I error increased—reaching up to 0.378 for some strategies. The last common visit strategy was the only approach that consistently preserved nominal error rates (0.025 ± 0.002). Regarding statistical power, all methods exhibited a loss of precision and increased bias under the alternative hypothesis as missingness increased. Multiple imputation partially recovered power and reduced bias but inflated the Type I error rate in scenarios with differential attrition. As generalized pairwise comparisons are increasingly used in pivotal clinical trials—and therefore in regulatory contexts—our findings provide practical guidance for selecting a robust primary analysis strategy and minimizing bias arising from incomplete data.

## 1 Introduction

Across a wide range of diseases, the gold standard for evaluating drug efficacy in randomized clinical trials is overall mortality [1, 2]. Longitudinal measures, however, could play a pivotal role in capturing a drug's impact on how a patient feels and functions during life [1]. These outcomes can offer valuable insights into the clinical meaningfulness and risk–benefit profile of new therapies. Consequently, multiple outcomes are often considered important in clinical trials [2, 3, 4]. Longitudinal measures may be prioritized when mortality requires long follow‐up, or when functional outcomes are equally relevant to understanding the clinical benefit. A common strategy is to combine longitudinal measures with survival into a single composite endpoint—either to improve statistical efficiency and the feasibility of trial conduct, or to better capture the full response to treatment in complex diseases that affect multiple domains simultaneously.

One composite approach gaining momentum is a non‐parametric method involving generalized pairwise comparisons [2, 5, 6], which allows for the assessment of multiple outcomes in either a prioritized or non‐prioritized manner [4]. The general concept involves comparing pairs of patients across two or more outcomes and determining which patient has the better result. Outcomes are typically prioritized by clinical relevance or event severity [5, 6], but can also be ranked based on patient preferences [7], or treated as equally important [8]. These strategies are extensions of the Mann–Whitney formulation of the Wilcoxon rank‐sum test. The prioritized variant was first proposed by Finkelstein and Schoenfeld in 1999 to combine mortality and longitudinal data [6]. Subsequent generalizations by Buyse and Pocock et al. led to the development of win statistics [5, 9], further formalizing this approach.

An example of the prioritized approach is the Combined Assessment of Function and Survival (CAFS) [10], a primary outcome for clinical trials in Amyotrophic Lateral Sclerosis (ALS). In this method, time to death is compared first within all possible patient pairs using a win‐loss rule, where patients who live longer are considered to “win.” If a comparison is tied, the pair is compared based on functional disability. All pairwise comparisons are subsequently summarized to estimate the probability that a patient in the treatment arm has a better outcome than a patient in the control arm. CAFS illustrates the flexibility of generalized pairwise comparisons, as it can accommodate virtually any type of outcome and is less restrictive than parametric or semi‐parametric alternatives [1, 2]. Moreover, it provides a robust solution to account for informative censoring in longitudinal measures due to death, helping to avoid situations where higher mortality in one arm could paradoxically yield better study outcomes [10].

**FIGURE 1:**
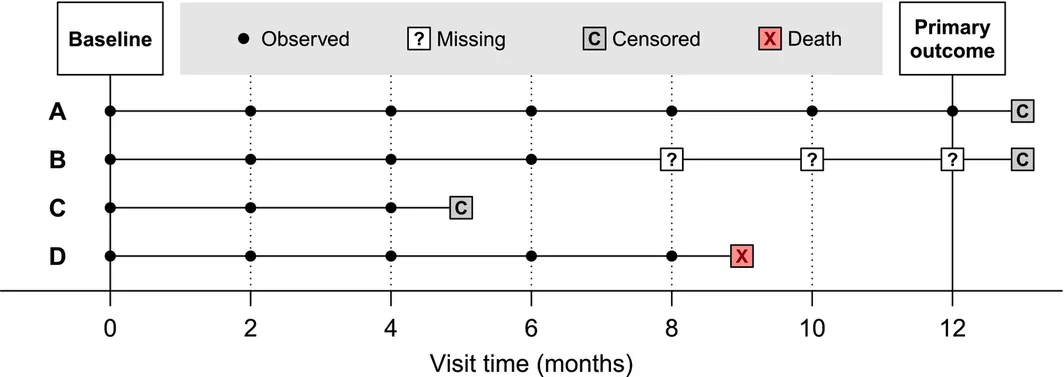
Time horizon for four hypothetical patients in a 12‐month clinical trial with bimonthly follow‐up measurements of physical functioning. The primary outcome is a composite endpoint of death and functional loss at 12 months. Patient A survives and has complete data. Patient D dies during follow‐up, with survival status known and longitudinal data truncated at death. Patient B has a known survival status at month 12 but missing longitudinal measurements. Patient C has missing longitudinal measurements and an unknown survival status at month 12.

Given these advantages, generalized pairwise comparisons are increasingly used as primary outcomes in pivotal clinical trials [11, 12]. In the case of ALS, the use of CAFS as a primary or key secondary outcome in clinical trials is even specifically recommended by the Food and Drug Administration (FDA) and the European Medicines Agency (EMA) [13, 14]. A common challenge, however, is the occurrence of missing data in studies of fixed duration. Missing data may arise solely in the longitudinal outcome, or may be accompanied by censoring of survival time before the planned study endpoint, as illustrated in Figure 1. In their original work, Finkelstein and Schoenfeld addressed missing data in the longitudinal marker by comparing outcomes at the earlier of a pair's follow‐up times (or the last common visit) [6]. Since then, various alternative strategies have been used. To illustrate, Table 1 provides an overview of different strategies used in ALS clinical trials to address missing data, where even within the regulatory context, multiple approaches have been applied [15, 16, 17].

**TABLE 1:** An overview of ALS clinical trials implementing the combined assessment of function and survival (CAFS).

Drug	Year	Time‐to‐event outcome (*survival*)	Longitudinal outcome (*ALSFRS‐R*)	Missing data strategy
Arimoclomol	2017	Death or PAV	Average slope over time	Comparison of the last observed slope
Arimoclomol	2024	Death or PAV	CFB	Comparison at the last common visit of a pair
CMN‐Au8	2023	Death	CFB	Comparison at the last common visit of a pair
Dexpramipexole	2013	Death	CFB	Comparison at the last common visit of a pair
Levosimendan	2021	Death	CFB	Multiple imputation of missing longitudinal scores
MSC‐NTF	2022	Death	CFB	Last rank carried forward
Ozanezumab	2017	Death	CFB	Comparison at the last common visit of a pair
PB‐TURSO	2022	Death or PAV	CFB	Comparison of the last observed change from baseline
Ravulizumab	2023	Death	Average slope over time	Comparison of the last observed slope
Tofersen	2022	Death	CFB	Multiple imputation of missing longitudinal scores

Abbreviations: ALSFRS‐*R*, Amyotrophic Lateral Sclerosis Function Rating Scale—Revised; CFB, change from baseline; PAV, permanent assisted ventilation.

How different missing data strategies impact the integrity of generalized pairwise comparison methods when combining longitudinal and time‐to‐event outcomes has received little attention in the literature [18, 19]. Specifically, it remains unclear whether each approach is unbiased, appropriately controls Type I error, or minimizes loss of efficiency. In this simulation study, therefore, we will evaluate and compare the bias and operating characteristics of different missing data strategies when applying a hierarchical endpoint based on generalized pairwise comparisons. The CAFS used in ALS clinical trials will serve as a case study, with the aim of generating recommendations for future trials.

## 2 Methods

### 2.1 Analytical Strategies

Our primary objective was to evaluate the impact of missing data on the Type I and Type II error rates of the CAFS, and the bias in the estimated treatment effect. The CAFS ranks patients through pairwise comparisons based on their survival time or the change from baseline in ALS functional rating scale (ALSFRS‐R) total score at the end of a fixed follow‐up period, typically 12 or 18 months. For each comparison, we determine whether a patient wins, loses, or ties with their comparator. By calculating the number of wins and losses for each patient, we can rank them based on survival time and change from baseline. In general, the patient who dies first receives the lowest rank, while the patient who survives with the least decline from baseline receives the highest. Ranks between treatment arms are compared using a Mann–Whitney *U* test, with the effect size expressed as a win probability. The win probability reflects the probability that a random patient in the treatment group has a better outcome compared to a random patient in the control group.

From a missing‐data perspective, death introduces informative truncation of the longitudinal ALSFRS‐R outcome, as post‐death measurements are unobserved due to a terminal event that depends on the underlying disease process. This is typically regarded as a form of Missing Not At Random (MNAR). The CAFS incorporates death directly into the composite endpoint, thereby redefining the estimand so that post‐death ALSFRS‐R measurements are not required and eliminating the need to define or impute post‐death functional outcomes. Nevertheless, additional non‐death‐related attrition and missing ALSFRS‐R data may occur. Censoring is typically assumed to be non‐informative, and the CAFS still requires an observed end‐of‐trial ALSFRS‐R outcome for censored patients. Several approaches have therefore been proposed to handle these incomplete observations (Table 1).

1. *Last observed change from baseline (CFB)*—This method ranks deceased patients first, followed by dropouts and completers based on their last observed change from baseline. This approach effectively assumes that the last observed ALSFRS‐R adequately represents the patient's subsequent disease course. Since ALS is a progressive disease, early dropout results in less observed decline from baseline compared to observed results when completing the study. In addition, it overlooks the fact that a dropout may have a censoring time earlier than a recorded death, meaning the dropout could theoretically have died before the observed death. Both factors thereby “reward” early dropout with a higher rank.
2. *Last observed slope—*This method ranks deceased patients first, followed by dropouts and completers, based on their last observed change from baseline divided by time since baseline. By standardizing change relative to time in the study, it yields a rate of change (slope) and improves upon the last observed CFB method. Nevertheless, this method still assumes that the future trajectory can be inferred from the observed data without explicitly modeling the missing data mechanism. Moreover, it does not account for cases where a dropout may have died before a recorded death in the trial. A simple adjustment is to compare slope estimates in cases where it is unclear whether a dropout survived beyond a deceased patient. In this adjusted approach, a deceased patient may “win” against a dropout based on their last observed slope estimate, potentially receiving a higher rank.
3. *Zero or tie allocation—*This method is similar to the adjusted slope approach, but for all comparisons in which follow‐up ALSFRS‐R data are missing, a tie (score of zero) is assigned [18]. By assigning ties whenever follow‐up data are unavailable, this approach assumes that missing follow‐up data provide no evidence in favor of either treatment. In this case, deaths are tied with patients who drop out. However, since dropouts can no longer lose a comparison (unlike deaths), they are always ranked higher than deceased patients.
4. *Hierarchical approach*—This approach ranks deaths first, followed by dropouts ordered by their last known assessment time, and then completers ordered by their change from baseline. This approach implicitly assumes that patients who discontinue follow‐up would have experienced worse functional outcomes than completers, an assumption resembling a MNAR‐type missing data mechanism. As a result, dropouts are ranked lower than completers, but this method does not account for the possibility that a dropout could have died before a patient who was recorded as dead during the trial. In addition, if a dropout had survived until the end of the trial, this approach assumes that their ALSFRS‐R score would have been lower than that of a completer.
5. *Multiple imputation—*This approach imputes missing ALSFRS‐R scores for dropouts using a multiple imputation strategy. Unlike the rule‐based approaches above, multiple imputation assumes that, conditional on the observed data included in the imputation model, missing ALSFRS‐R values are Missing At Random (MAR). Moreover, as censored survival time was not imputed in previous studies [20, 21], it assumes that dropouts survived until the end of the study, meaning they are always ranked higher than patients who died.
6. *Last‐rank‐carried‐forward—*In this method, also known as ALS/SURV [22], ranking occurs at each visit, and rank percentiles are calculated based on the number of patients ranked at each visit. For patients who drop out, the last observed percentile rank is carried forward to obtain the final ranking at the end of the study. This approach avoids imputing missing ALSFRS‐R scores but assumes that a patient's relative rank remains unchanged after dropout, irrespective of subsequent disease progression. Of note, patients who die are always included in the ranking at each visit, whereas dropouts are only included until their last observed ALSFRS‐R score. As a result, any information about a dropout remaining alive after their last observed ALSFRS‐R score is ignored.
7. *Last‐common‐visit—*This approach was originally described by Finkelstein and Schoenfeld and for the CAFS [6, 10], where patients are compared based on their last common visit. A patient who dies loses to a dropout if the dropout is censored after the death time. However, if the dropout is censored before the death time, the pair is compared based on their last common visit. This approach avoids any need to specify or impute ALSFRS‐R values beyond the observed follow‐up and implicitly conditions inference on the observed follow‐up window, assuming that the observed trajectories within this shared window are sufficient to determine the relative ordering of patients. In this approach, patients who die may win against a dropout and could therefore be ranked higher. Moreover, unlike the last‐rank‐carried‐forward approach, the censoring time is not ignored. This means that all dropouts with missing ALSFRS‐R scores but known to be alive at the end of the trial will win against patients who died during follow‐up.

### 2.2 Individual Patient Data

A simulation study was conducted to assess the impact of these approaches on the operational characteristics of the CAFS. Simulation parameters for generating correlated longitudinal (ALSFRS‐R) and time‐to‐event (survival) data were based on the PRECISION ALS study [23]. In brief, PRECISION ALS was a population‐based study conducted at seven sites in Europe. To ensure comparability with a typical Phase III trial population, patients were excluded if they had a symptom duration—defined as the time between symptom onset and the first ALSFRS‐R assessment—exceeding 36 months, a %predicted vital capacity below 60%, or an age above 80 years. This enriched the sample for patients with earlier disease stage and resulted in a trial‐eligible cohort of 4927 patients who contributed 24 564 ALSFRS‐R measurements over a follow‐up period of 7581 person‐years. A joint modeling framework was employed to estimate the population's rate of decline in the ALSFRS‐R total score, the variability in ALSFRS‐R trajectories between patients, and the relationship between ALSFRS‐R and survival. The data processing steps, model selection procedures and model output are described elsewhere [23]; the simulation parameters used in this study were taken directly from the optimal model identified in the previous study, with all parameter values reproduced as reported therein (e‐Table 1 in Supporting Information).

### 2.3 Data Generating Mechanism

We simulated ALSFRS‐R follow‐up measurements (the *longitudinal process*) alongside conditional survival data (the *time‐to‐event process*). The latent longitudinal process $m_{i}\left(t\right)$ to describe ALSFRS‐R decline since initial assessment over time *t* (in years) in treatment group $\omega_{i}$ (0 for control, 1 for treatment) for the *i*th patient was given by

$$\text{ALSFRS}-R_{i}\left(t\right)=m_{i}\left(t\right)+\varepsilon_{i}\left(t\right)$$

(1) $$\begin{array}{ll} m_{i}\left(t\right) & =\left(\beta_{0}+\mu_{0i}\right)+\left(\beta_{1}+\mu_{1i}\right)\cdot t+\left(\beta_{2}+\mu_{2i}\right)\cdot t^{2}\\ & \;+\beta_{3}\cdot \omega_{i}\cdot t+\beta_{4}\cdot \omega_{i}\cdot t^{2}, \end{array}$$

where $\beta_{0-4}$ are the fixed effects, $\mu_{0-2}$ the random effects for patient *i*, and $\varepsilon_{i}$ the error terms. The time trajectory follows a quadratic curvature, where $\beta_{0}$ represents the population‐average ALSFRS‐R score at baseline, $\beta_{1}$ the population‐average rate of linear change (decline), and $\beta_{2}$ the population‐average curvature, reflecting acceleration or deceleration of the rate of decline over time. Patient‐specific parameters $(\beta_{0}+\mu_{0i}$), $(\beta_{1}+\mu_{1i}$) and ($\beta_{2}+\mu_{2i}$) were generated by sampling from a multivariate normal distribution. ALSFRS‐R scores were obtained by adding independent random error (ε(t)), drawn from a normal distribution with a mean of 0 and a standard deviation of $\sigma_{e}$. Scores were rounded to the nearest integer and constrained within the range of 0 to 48. Time *t* ranged from baseline (*t* = 0) to 1 year, with assessments every 2 months. Subsequently, conditional survival times were simulated using a Weibull distribution, where the hazard function depended on both the current value of $m_{i}\left(t\right)$ and its slope at time *t*. The hazard function was given by

(2) $$h_{i}\left(t\right)=p\cdot t^{p-1}\cdot e^{\gamma_{0}+\gamma_{1}\cdot \omega_{i}+\alpha_{1}\cdot m_{i}\left(t\right)+\alpha_{2}\cdot m_{i}^{\prime }\left(t\right)},$$

where

$$m_{i}^{\prime }\left(t\right)=\frac{d}{\mathrm{dt}}m_{i}\left(t\right)=\left(\beta_{1}+\mu_{1i}\right)+2\cdot \left(\beta_{2}+\mu_{2i}\right)\cdot t+\beta_{3}\cdot \omega_{i}+2\cdot \beta_{4}\cdot \omega_{i}\cdot t,$$

and $e^{\gamma_{0}}$ and *p* the Weibull scale and shape parameters, respectively, $\alpha_{1}$ the log‐hazard ratio of the current value of $m_{i}\left(t\right)$, and $\alpha_{2}$ the log‐hazard ratio of the slope of $m_{i}\left(t\right)$ at time *t*. In this framework, treatment may have a *direct* effect on survival, independent of the ALSFRS‐R, through $\gamma_{1}$ (Equation 2). Additionally, treatment may exert an *indirect* effect on survival by moderating the ALSFRS‐R slope via the terms $\beta_{3}$ and $\beta_{4}$ (Equation 1) [1]. Only when $\alpha_{1}=\alpha_{2}=0$ does $\text{ALSFRS}-R_{i}\left(t\right)$ become independent of survival time, resulting in a MAR‐type mechanism for ALSFRS‐R measurements after death. Otherwise, the time‐to‐event process depends on the unobserved latent ALSFRS‐R trajectory and its rate of decline (i.e., $m_{i}\left(t\right)\;\text{and}\;m_{i}^{\prime }\left(t\right)$), resulting in a MNAR‐type mechanism for post‐mortem ALSFRS‐R measurements. In our simulation study, survival time (*t*) was simulated using the inverse transformation method, where survival probabilities were drawn from a Uniform (0,1) distribution and transformed via the probability integral transformation to obtain *t*. Simulated survival times were censored at 1 year. The ALSFRS‐R scores of subjects who died were recoded as missing after the event. Additionally, the interval between death and the last observed ALSFRS‐R score was simulated by sampling from a log‐normal distribution (mean = −0.715, SD = 0.637). Visits falling within this interval were recorded as missing, reflecting that ALSFRS‐R assessments are typically not performed immediately before death. These values were based on data from a completed clinical trial [24].

Finally, we considered two additional missing data mechanisms. First, complete loss to follow‐up (LTFU) could occur, resulting in missing ALSFRS‐R scores and an unknown vital status at the end of follow‐up. Second, ALSFRS‐R scores could be missing while the vital status remained known, as survival information is often obtainable without direct patient contact. Both LTFU and missing ALSFRS‐R times were sampled independently from exponential distributions with baseline yearly attrition probabilities $\delta_{0,\;\text{LTFU}}$ and $\delta_{0,\;\text{Missing}}$, respectively. To simulate differential attrition rates between treatment arms and introduce dependency between baseline disease severity and the risk of attrition, individual‐specific attrition rates were defined as:

$$\lambda_{\text{LTFU},i}=-\log\left(1-\frac{\delta_{0,\;\text{LTFU}}}{100}\right)\times \exp\left[\log\left(\delta_{1,\;\text{LTFU}}\right)\omega_{i}+\log\left(\delta_{2}\right)\mu_{0i}\right]$$

and

$$\lambda_{\text{Missing},i}=-\log\left(1-\frac{\delta_{0,\;\text{Missing}}}{100}\right)\times \exp\left[\log\left(\delta_{1,\;\text{Missing}}\right)\omega_{i}+\log\left(\delta_{2}\right)\mu_{0i}\right]$$

where $\delta_{0,\text{LTFU}}$ and $\delta_{0,\text{Missing}}$ denote the baseline yearly attrition probabilities (%), $\delta_{1,\text{LTFU}}$ and $\delta_{1,\text{Missing}}$ denote the hazard ratios associated with treatment allocation, and $\delta_{2}$ denotes the hazard ratio for the random intercept (i.e., the relative baseline severity compared to the population average). Individual attrition times were subsequently generated using inverse transform sampling:

$$T_{\text{LTFU},i}=\frac{-\log\left(U_{1i}\right)}{\lambda_{\text{LTFU},i}}$$

and,

$$T_{\text{Missing},i}=\frac{-\log\left(U_{2i}\right)}{\lambda_{\text{Missing},i}}$$

where $U_{1i}$ and $U_{2i}∼U\left(0,1\right).$ This specification allows for a range of missing data mechanisms. Differences in $\delta_{1}$ induce differential attrition between treatment arms, allowing assessment of the analytical strategies under asymmetric dropout patterns. When $\delta_{1}$ = 1.0 and $\delta_{2}=1.0$, attrition is independent of treatment and disease severity, and corresponds to a Missing Completely at Random (MCAR)‐type mechanism. When $\delta_{2}\neq 1.0$, attrition depends on the patient‐specific random intercept, introducing a dependency between baseline disease severity and the probability of missingness. Because the measurement error variance is relatively small compared with the between‐patient variability in this simulation study (e‐Table 1 in Supporting Information), the observed baseline ALSFRS‐R score is a relatively accurate proxy for the underlying patient‐specific random intercept. As a result, the mechanism is close to one in which missingness depends on observed characteristics, although it does not strictly satisfy the MAR assumption. There is no MNAR mechanism introduced for loss to follow‐up and missing ALSFRS‐R time.

**FIGURE 2:**
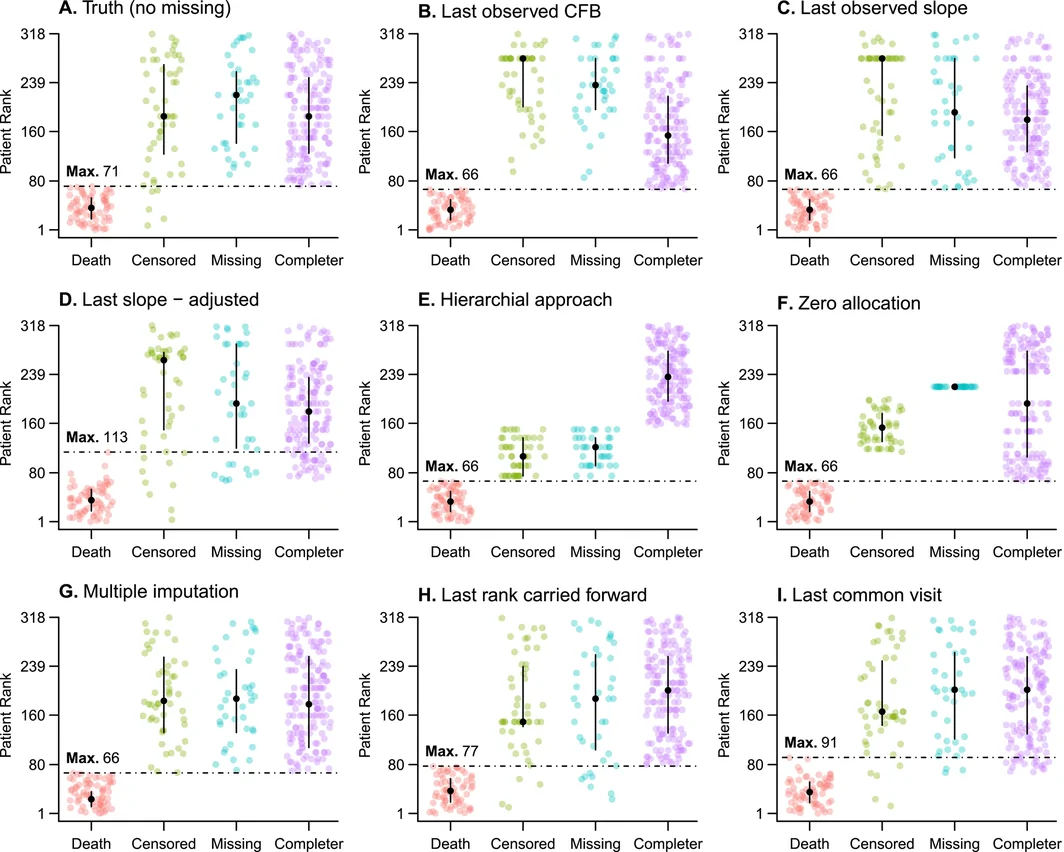
Impact of the missing data strategy on patient ranking in a hierarchical composite outcome of mortality and function. For a single simulated dataset of *N* = 318, the ranks of the individual patients were calculated according to the different strategies. Ranks were stratified according to missing data status at the end of the study (month 12). Censored = patients who are lost‐to‐follow‐up (LTFU), having missing ALSFRS‐R scores and unknown vital status at end of the study; Missing = patients with only missing ALSFRS‐R scores, but known vital status at end of follow‐up. The black dashed line marks the upper rank assigned to deceased patients.

Finally, Figure 2 presents the ranks for one simulated trial, illustrating the impact of the different analytical strategies on patient ranks, with $\delta_{0,\;\text{LTFU}}$ = 20% and $\delta_{0,\;\text{Missing}}$ = 20%, reflecting a 20% attrition rate in LTFU and ALSFRS‐R scores after 1 year.

### 2.4 Simulation Design

We employed a full factorial simulation design, considering a total of 144 missing data scenarios under the null hypothesis with different parameter settings for $\delta_{0,\text{LTFU}}$, $\delta_{0,\text{Missing}},\delta_{1,\text{LTFU}}$, and $\delta_{1,\text{Missing}}$ (Table 2). Values were based on commonly reported attrition rates in previous ALS clinical trials [25]. Moreover, we considered a scenario in which attrition rates were either independent or depended on baseline severity via parameter $\delta_{2}$. All scenarios were evaluated for a hypothetical 1:1 randomized study with bimonthly visits over 12 months. Sample size was based on a common 25% reduction in disease progression rate and hazard (i.e., $\beta_{3}=-0.25\cdot \beta_{1},\beta_{4}=-0.25\cdot \beta_{2},\gamma_{1}=\log\left(1-0.25\right),$ resulting in a win probability of 0.591). Based on simulations, a sample size of 318 patients resulted in 80.3% power (95% CI: 79.6% to 81.0%, e‐Figure 1 in Supporting Information). Overall, when assuming no censoring other than death, these settings resulted in a simulated 6‐month survival of 92.6% and a 12‐month survival of 81.1% in the placebo arm, with an average ALSFRS‐R progression rate over 12 months of −1.06 points per month, consistent with values typically observed in ALS clinical trials [24].

**TABLE 2:** Overview of simulation factors, symbols, and scenarios.

Simulation factor	Symbol	Scenario
Yearly loss‐to‐follow‐up rate for vital status	$\delta_{0,\;\text{LTFU}}$	0%, 10%
Yearly missing data rate in ALSFRS‐R	$\delta_{0,\text{Missing}}$	0%, 10%, 20%, 30%
Hazard ratio λ_LTFU_ placebo versus treatment	$\delta_{1,\text{LTFU}}$	0.50, 1.00, 2.00
Hazard ratio λ_Missing_ placebo versus treatment	$\delta_{1,\text{Missing}}$	0.50, 1.00, 2.00
Hazard ratio λ_LTFU_ and λ_Missing_ baseline severity	$\delta_{2}$	0.93, 1.00

Abbreviations: ALSFRS‐*R*, Revised ALS Function Rating Scale; LTFU, loss‐to‐follow‐up.

In addition, we evaluated three scenarios under the alternative hypothesis: (1) a common 25% reduction in both the disease progression rate and hazard, (2) a 25% reduction in the disease progression rate with no effect on survival, and (3) a 50% reduction in the hazard with no effect on function. All alternative‐hypothesis scenarios were evaluated under a balanced missing data setting (i.e., $\delta_{1}$ = 1.0), assuming a baseline loss‐to‐follow‐up probability of 10% ($\delta_{0,\text{LTFU}}$ = 10%), loss to follow‐up dependent on baseline disease severity ($\delta_{2}$ = 0.93), and varying the yearly probability of additional missing ALSFRS‐R scores ($\delta_{0,\text{Missing}})$ from 0% to 30%.

### 2.5 Number of Iterations and Random Sampling

For each missing data scenario, independent datasets were generated; independence of scenarios was assured by using a different starting seed for each scenario. All statistical strategies were applied to the same generated dataset to remove between‐simulation variability. For the multiple imputation approach, a two‐level multiple imputation by chained equations generated five imputed datasets for missing ALSFRS‐R scores. Imputation followed a fully conditional specification with homogeneous residual variances, using the “2l.pan” method in the *mice* package (version 3.17.0, 2024, van Buuren S) [26]. Imputations were generated separately for each randomized group [27]. The corresponding imputation model incorporated all available ALSFRS‐R data, with fixed effects for baseline ALSFRS‐R score, survival time (transformed via the Nelson‐Aalen estimator of the cumulative hazard function), and vital status. A random effect accounted for visit time (in years) by patient. Imputed values were constrained to integers within the 0–48 range. Rankings were determined within each imputed dataset, and results were combined using Rubin's rules.

In case of a failed iteration (due to convergence failure of the imputation algorithm), the sample was disregarded, and a new sample was generated. The number of failed iterations was recorded, occurring in 0.009% of the iterations due to imputation model convergence issues and not being related to a specific scenario. For each scenario, we estimated the Type I and II error rates by calculating the proportion of simulation samples in which the null hypothesis of no effect was rejected. In addition, bias in the win probability was determined. Based on the variance of a proportion, 15 000 iterations per scenario were deemed necessary to obtain a 95% accuracy interval around a 0.0250 one‐sided Type I error ranging from 0.0225 to 0.0275. Random numbers were produced by the L'Ecuyer‐CMRG algorithm using R version 4.5.1 (2025‐06‐13).

## 3 Results

**FIGURE 3:**
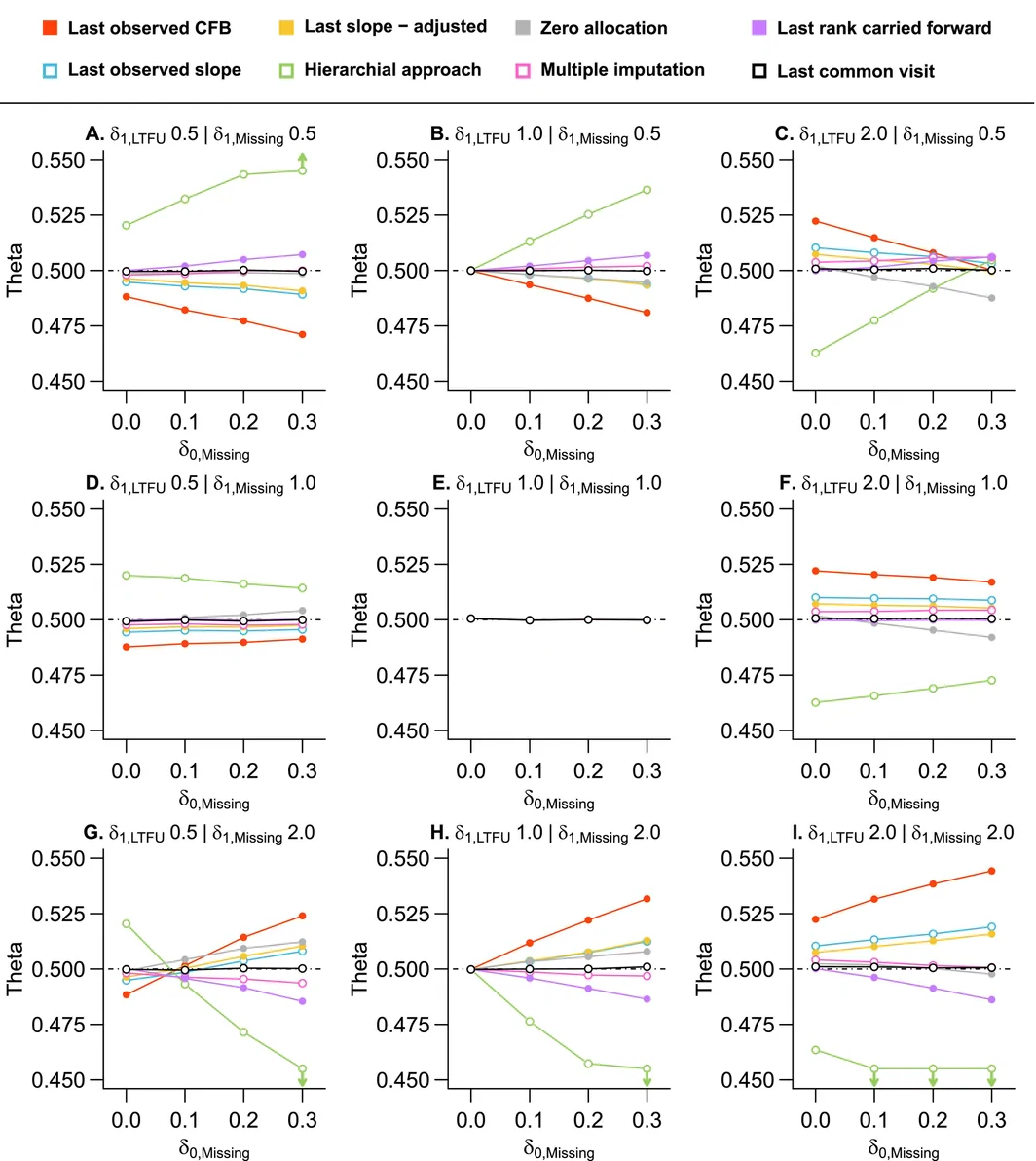
Bias under the null hypothesis in the CAFS win probability across different analytical strategies. Bias in the win probability (theta) was determined for each strategy under the null hypothesis (win probability of 0.5). The panels reflect the different simulation scenarios provided in Table 2; *δ*_1,LTFU_ reflects the hazard ratio of the censoring time for survival (i.e., both ALSFRS‐R and survival time are missing); *δ*_1,Missing_ reflects the hazard ratio of the censoring time for ALSFRS‐R (i.e., only ALSFRS‐R is missing), with values less than 1 indicating there is less censoring in the treatment arm. The baseline ALSFRS‐R censor rate (*δ*_0,Missing_) is depicted on the *x*‐axis, the baseline survival censor rate (*δ*_0,LTFU_) is fixed at 10%. Results exceeding the plotting range are indicated with arrows; the actual values are 0.553 (panel A), 0.454 (panel G), 0.443 (panel H), and 0.445, 0.431, and 0.422 (panel I).

**FIGURE 4:**
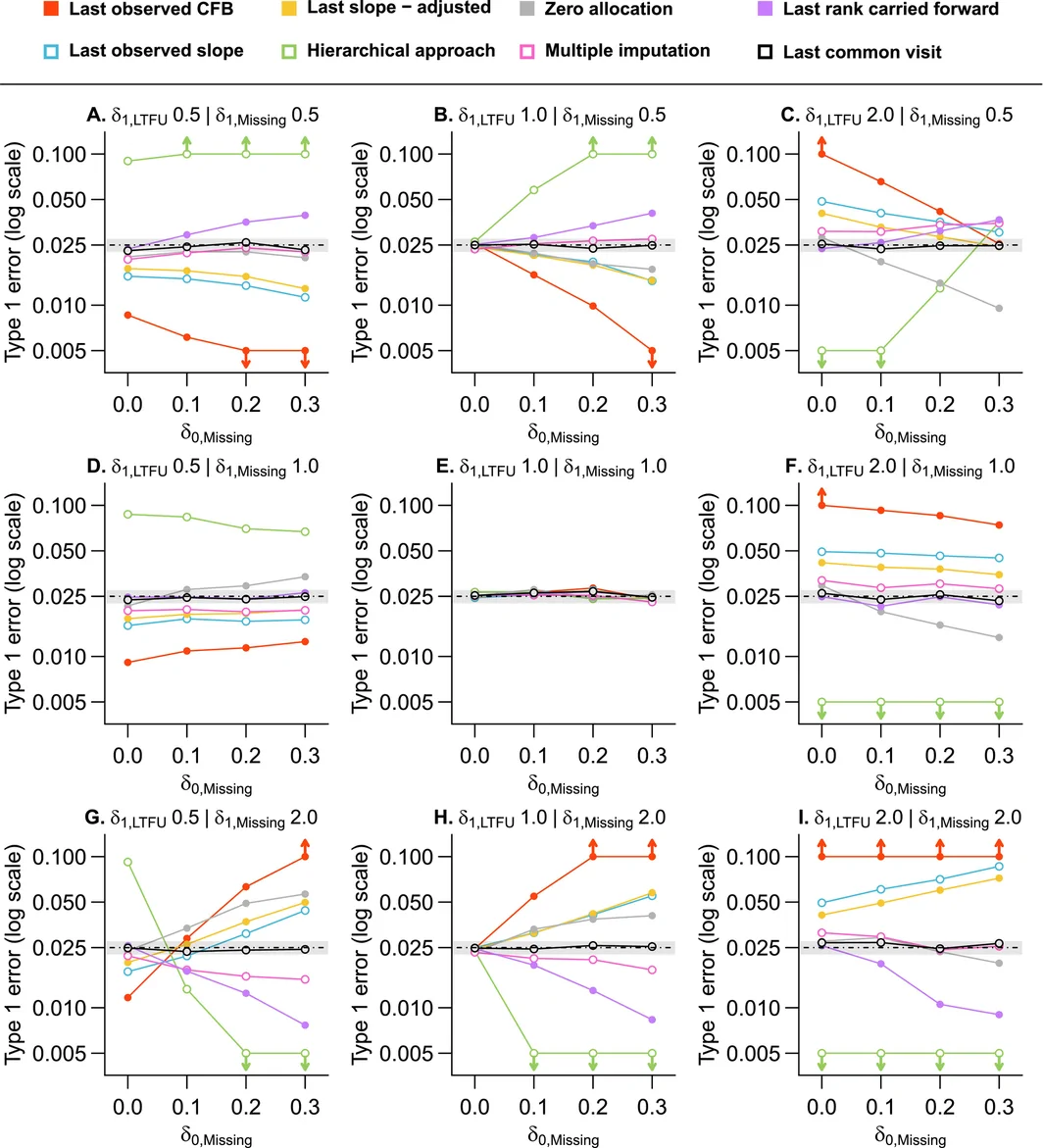
Empirical one‐sided Type 1 error of each analytical strategy. The one‐sided Type I error was estimated empirically as the proportion of simulation replicates in which the null hypothesis of no treatment effect was rejected, under the scenario where treatment had no effect on either function or survival. The panels correspond to the various simulation scenarios shown in Figure 3. The dashed horizontal line indicates the nominal Type I error rate (0.025), and the grey shaded area represents the expected range due to simulation variability (0.0225 to 0.0275). Results exceeding the plotting range are indicated with arrows; numerical results are presented in Table 3.

In total, we evaluated 144 simulation scenarios under the null hypothesis and show the performance of each analytical strategy in terms of bias (Figure 3) and Type I error control (Figure 4). Results are based on a subset of 36 scenarios with a baseline loss‐to‐follow‐up probability of 10% ($\delta_{0,\text{LTFU}}$ = 10%) and loss to follow‐up dependent on baseline disease severity ($\delta_{2}$ = 0.93). Results from the remaining scenarios are provided in the Supporting Information. Overall, all analytical strategies were unbiased under the null hypothesis and maintained appropriate Type I error control when missing data and censoring rates were balanced across treatment arms (Figures 3, 4, Panel E). Here, $\delta_{1,\text{LTFU}}$ reflects the hazard ratio of the censoring time for survival (i.e., both ALSFRS‐R and survival time are missing); $\delta_{1,\text{Missing}}$ reflects the hazard ratio of the censoring time for ALSFRS‐R (i.e., only ALSFRS‐R is missing), with values less than 1 indicating there is less censoring in the treatment arm. In imbalanced scenarios, that is, either $\delta_{1,\text{Missing}}$ ≠ 1.0 or $\delta_{1,\text{LTFU}}$ ≠ 1.0, substantial differences emerged. Type I error rates reached as high as 0.378 (95% CI: 0.379 to 0.386) for the hierarchical approach and 0.276 (95% CI: 0.269 to 0.283) for the last observed change from baseline approach (Table 3).

**TABLE 3:** Empirical one‐sided Type 1 error of each analytical strategy.

Yearly missing data in ALSFRS‐R $\;\left(\delta_{0,\text{Missing}}\right)$	Hazard ratio for loss‐to‐follow‐up $\;(\delta_{1,\text{LTFU}}$)	Hazard ratio for missing data in ALSFRS‐R $\;\left(\delta_{1,\text{Missing}}\right)$	Last observed CFB	Lastobserved slope	Last slope – adjusted	Hierarchical approach	Zero allocation	Multiple imputation	Last rank carried forward	Last common visit
0%	1.0	1.0	0.0248 (±0.0013)	0.0245 (±0.0013)	0.0248 (±0.0013)	0.0267 (±0.0013)	0.0251 (±0.0013)	0.0252 (±0.0013)	0.0253 (±0.0013)	0.0253 (±0.0013)
30%	1.0	1.0	0.0238 (±0.0012)	0.0241 (±0.0013)	0.0241 (±0.0013)	0.0243 (±0.0013)	0.0255 (±0.0013)	0.0229 (±0.0012)	0.0247 (±0.0013)	0.0246 (±0.0013)
0%	0.5	1.0	0.0091 (±0.0008)	0.0160 (±0.0010)	0.0178 (±0.0011)	0.0872 (±0.0023)	0.0216 (±0.0012)	0.0201 (±0.0011)	0.0247 (±0.0013)	0.0237 (±0.0012)
30%	0.5	1.0	0.0125 (±0.0009)	0.0175 (±0.0011)	0.0204 (±0.0012)	0.0669 (±0.0020)	0.0337 (±0.0015)	0.0202 (±0.0011)	0.0264 (±0.0013)	0.0249 (±0.0013)
0%	2.0	1.0	0.1005 (±0.0025)	0.0493 (±0.0018)	0.0417 (±0.0016)	0.0014 (±0.0003)	0.0295 (±0.0014)	0.0319 (±0.0014)	0.0249 (±0.0013)	0.0262 (±0.0013)
30%	2.0	1.0	0.0741 (±0.0021)	0.0449 (±0.0017)	0.0347 (±0.0015)	0.0025 (±0.0004)	0.0133 (±0.0009)	0.0281 (±0.0013)	0.0219 (±0.0012)	0.0233 (±0.0012)
0%	1.0	0.5	0.0256 (±0.0013)	0.0248 (±0.0013)	0.0245 (±0.0013)	0.0263 (±0.0013)	0.0252 (±0.0013)	0.0235 (±0.0012)	0.0252 (±0.0013)	0.0250 (±0.0013)
30%	1.0	0.5	0.0043 (±0.0005)	0.0145 (±0.0010)	0.0145 (±0.0010)	0.2015 (±0.0033)	0.0173 (±0.0011)	0.0274 (±0.0013)	0.0406 (±0.0016)	0.0249 (±0.0013)
0%	0.5	0.5	0.0086 (±0.0008)	0.0155 (±0.0010)	0.0175 (±0.0011)	0.0899 (±0.0023)	0.0209 (±0.0012)	0.0201 (±0.0011)	0.0235 (±0.0012)	0.0229 (±0.0012)
30%	0.5	0.5	0.0020 (±0.0004)	0.0113 (±0.0009)	0.0129 (±0.0009)	0.3780 (±0.0040)	0.0206 (±0.0012)	0.0225 (±0.0012)	0.0393 (±0.0016)	0.0232 (±0.0012)
0%	2.0	0.5	0.1003 (±0.0025)	0.0486 (±0.0018)	0.0405 (±0.0016)	0.0005 (±0.0002)	0.0280 (±0.0013)	0.0308 (±0.0014)	0.0237 (±0.0012)	0.0254 (±0.0013)
30%	2.0	0.5	0.0255 (±0.0013)	0.0303 (±0.0014)	0.0243 (±0.0013)	0.0355 (±0.0015)	0.0095 (±0.0008)	0.0348 (±0.0015)	0.0367 (±0.0015)	0.0247 (±0.0013)
0%	1.0	2.0	0.0246 (±0.0013)	0.0249 (±0.0013)	0.0245 (±0.0013)	0.0237 (±0.0012)	0.0234 (±0.0012)	0.0233 (±0.0012)	0.0246 (±0.0013)	0.0248 (±0.0013)
30%	1.0	2.0	0.1674 (±0.0030)	0.0550 (±0.0019)	0.0577 (±0.0019)	0.0001 (±0.0001)	0.0407 (±0.0016)	0.0178 (±0.0011)	0.0083 (±0.0007)	0.0254 (±0.0013)
0%	0.5	2.0	0.0117 (±0.0009)	0.0173 (±0.0011)	0.0199 (±0.0011)	0.0921 (±0.0024)	0.0238 (±0.0012)	0.0221 (±0.0012)	0.0257 (±0.0013)	0.0249 (±0.0013)
30%	0.5	2.0	0.1081 (±0.0025)	0.0440 (±0.0017)	0.0497 (±0.0018)	0.0007 (±0.0002)	0.0565 (±0.0019)	0.0154 (±0.0010)	0.0077 (±0.0007)	0.0243 (±0.0013)
0%	2.0	2.0	0.1023 (±0.0025)	0.0495 (±0.0018)	0.0411 (±0.0016)	0.0014 (±0.0003)	0.0275 (±0.0013)	0.0313 (±0.0014)	0.0257 (±0.0013)	0.0269 (±0.0013)
30%	2.0	2.0	0.2755 (±0.0036)	0.0862 (±0.0023)	0.0720 (±0.0021)	0.0000 (±0.0000)	0.0197 (±0.0011)	0.0255 (±0.0013)	0.0090 (±0.0008)	0.0267 (±0.0013)

*Note:* The one‐sided Type I error was estimated empirically as the proportion of simulation replicates in which the null hypothesis of no treatment effect was rejected, under the scenario where treatment had no effect on either function or survival. The baseline survival censoring rate ($\delta_{0,\text{LTFU}}$) is fixed at 10%, with the probability of censoring depending on baseline disease severity ($\delta_{2}$ = 0.93). Values in brackets represent the Monte Carlo standard error.

Abbreviations: ALSFRS‐*R*, Revised ALS Function Rating Scale; LTFU, loss‐to‐follow‐up.

The last common visit approach was the only strategy that consistently controlled the Type I error across all scenarios and emerged as the most reliable method, with values ranging from 0.0229 (95% CI: 0.0205 to 0.02529) to 0.0270 (95% CI: 0.0244 to 0.0296) and an average Type I error of 0.0249 (Figure 4). The second‐best performing strategy was multiple imputation (range: 0.0154 to 0.0348), followed by the last rank carried forward approach (range: 0.0077 to 0.0406). Interestingly, the last rank carried forward approach was primarily affected in scenarios with imbalanced missing ALSFRS‐R data (i.e., $\delta_{1,\text{Missing}}$ ≠ 0). In contrast, it performed similarly to the last common visit approach even when censoring was imbalanced between treatment arms (i.e., $\delta_{1,\text{LTFU}}$ ≠ 0) and the overall rate of missing ALSFRS‐R data increased (i.e., $\delta_{0,\text{Missing}}$ > 0). Notably, the zero‐allocation approach—alongside the last common visit and last rank carried forward approach—was the only method that remained unbiased under the null hypothesis and controlled the Type I error in scenarios without additional ALSFRS‐R missing data (i.e., $\delta_{0,\text{Missing}}$ = 0). Results were similar when there was no association between baseline disease severity and censoring probability (Supporting Information).

**FIGURE 5:**
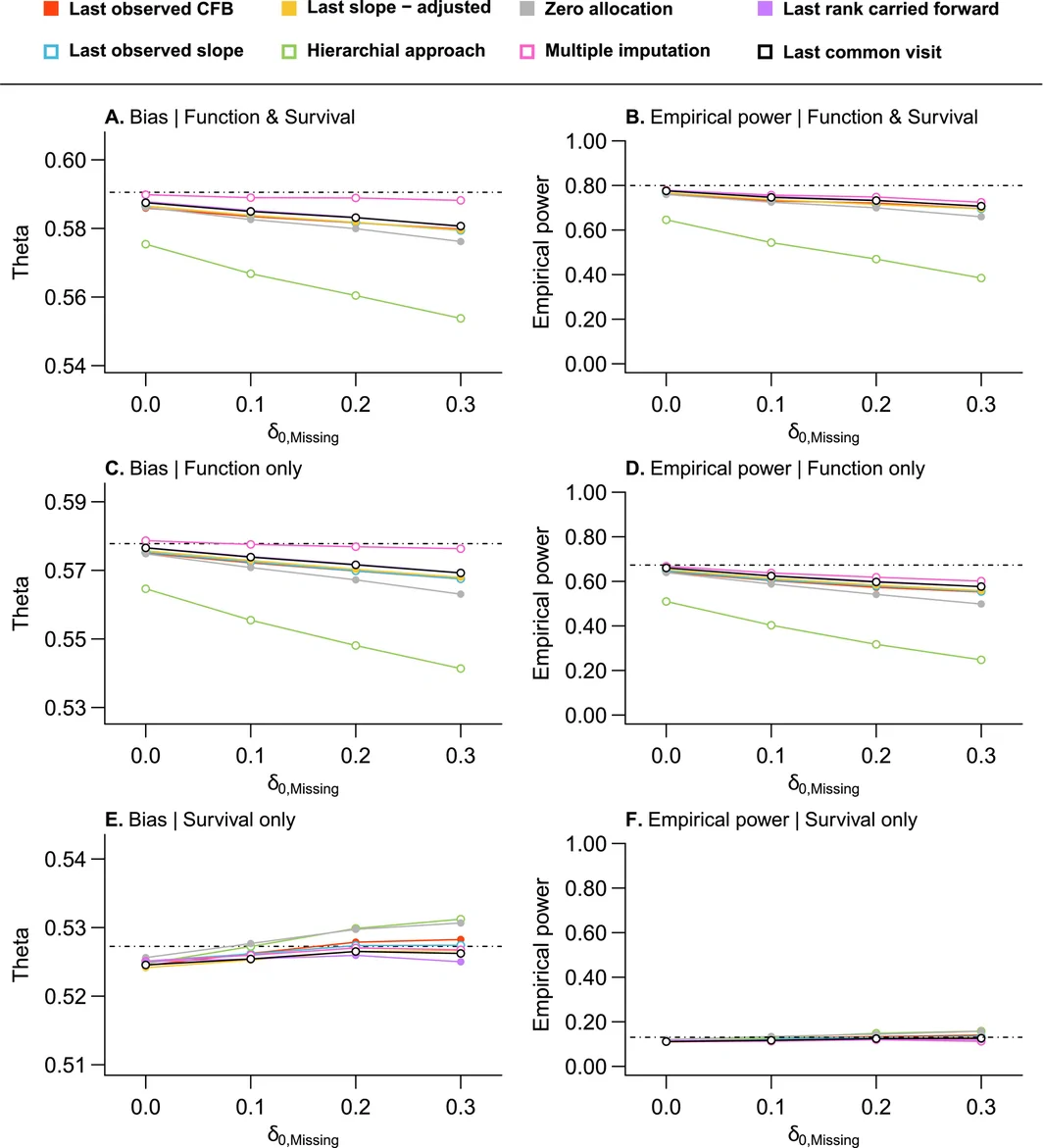
Bias and empirical statistical power of each analytical strategy under the alternative hypothesis. Bias in the win probability and statistical power were estimated for each strategy under three treatment scenarios: (1) a combined effect on function and survival (panels A–B), (2) an effect on function only (panels C–D), and (3) an effect on survival only (panels E–F). Statistical power was estimated empirically as the proportion of simulation replicates in which the null hypothesis of no treatment effect was rejected. The baseline censor rate (*δ*_0,LTFU_) was fixed at 10% and there is no differential missingness between treatment groups (i.e., *δ*_1,LTFU_ = 1.0 & *δ*_1,Missing_ = 1.0). The dashed horizontal line indicates the expected effect size and power when there is no missing and censoring.

Figure 5 depicts the bias and statistical power under three alternative hypothesis scenarios when dropout was balanced across treatment arms: (1) a treatment effect on both function and survival, (2) a treatment effect on only function, and (3) a treatment effect on only survival. In the scenarios with a treatment effect on function, all approaches showed increasing bias in the effect size and a loss of precision as ALSFRS‐R missingness increased. Even without any additional missing ALSFRS‐R data, the effect size based on the last common visit approach was biased due to the censoring of survival time (i.e., $\delta_{0,\text{LTFU}}$ = 10%). Of note, the multiple imputation approach led to the least biased estimates under the alternative hypothesis.

Interestingly, when treatment affects only survival (Figure 5, Panel E), estimated effect sizes tend to increase as the proportion of missing ALSFRS‐R data increases. Because no treatment effect is present on the functional component, increasing missingness reduces its contribution to the pairwise comparisons, thereby increasing the relative contribution of survival and resulting in less attenuation of the overall effect size [28]. In the most extreme case, in which all ALSFRS‐R data are missing, the CAFS would effectively be determined by survival alone, yielding an effect size closely related to that from a survival‐only analysis (e.g., a Cox proportional hazards model) [9]. In addition, patients in the treatment arm survive longer, resulting in a relatively larger proportion of missing ALSFRS‐R scores than in the control arm and consequently an imbalance in missing data between treatment groups, which may further contribute to differences between approaches. In the absence of censoring (i.e., $\delta_{0,\text{LTFU}}$ = 0%), all methods exhibited positive bias except the last rank carried forward approach (e‐Figure 4 in Supporting Information), because this method ignores information that a participant remained alive after their last observed ALSFRS‐R score.

In the scenario where there is both an effect on function and survival, statistical power for the last common visit approach declined from 77.6% (95% CI: 76.9 to 78.2) when $\delta_{0,\text{Missing}}$ = 0 to 70.7% (95% CI: 70.0 to 71.4) when $\delta_{0,\text{Missing}}$ = 0.3. This implies that, at $\delta_{0,\text{Missing}}$ = 0.3, sample sizes would need to be increased by 17.7% to maintain equivalent power. Under these conditions, the multiple imputation approach offered a modest power advantage over the last common visit approach—72.5% versus 70.7% at $\delta_{0,\text{Missing}}$ = 0.3—translating to a 4.2% difference in required sample size. When $\delta_{0,\text{Missing}}$ = 0, the statistical power was 77.6% for the last common visit approach and 76.0% for the zero‐allocation approach. Thus, although both methods are unbiased under the null hypothesis and maintain appropriate Type I error control in these settings, comparing patients with censored survival data using their last observed ALSFRS‐R score provides greater precision than treating such comparisons as ties.

## 4 Discussion

In this simulation study, we have evaluated how different missing data strategies affect the operating characteristics of generalized pairwise comparisons that combine overall survival with a longitudinal outcome in randomized clinical trials with a fixed follow‐up duration. Across all scenarios, comparing pairs of patients at their last common visit was the only method that consistently maintained nominal Type I error and produced unbiased estimates under the null hypothesis. Under the alternative hypothesis, all strategies demonstrated increasing bias in the treatment effect estimate and reduced precision as the proportion of missing longitudinal data increased. Multiple imputation provided a modest gain in power but inflated Type I error when vital status was unknown. As generalized pairwise comparisons are increasingly being used in pivotal clinical trials [29]—and thus in regulatory contexts—our findings offer practical guidance for selecting a robust primary analysis strategy and minimizing bias due to incomplete data.

Although the impact of censoring on generalized pairwise comparisons and the resulting bias in outcomes is well recognized [30, 31], most prior work has focused on time‐to‐event endpoints. Less attention has been given to missing data mechanisms occurring in longitudinal outcomes with repeated observations in fixed‐duration studies. Our study addressed this gap by providing a comprehensive, practical overview of strategies to handle missing data in longitudinal outcomes within the generalized pairwise comparison framework. One of these strategies has been to allocate a tie or zero, a method originally proposed by Gehan in 1965 [32]. While several studies have demonstrated the associated loss of precision, others have highlighted the potential bias this introduces in the treatment effect estimate under the alternative hypothesis [33, 34, 35]. In line with previous work [36, 37], we further show that assigning zeros may inflate the Type I error rate when missing data are imbalanced across treatment arms. As illustrated in Figure 2, this occurs because patients who drop out can no longer lose, unlike those who die, and therefore tend to receive a higher average rank.

Comparing patients at their last common visit or up to a shared length of follow‐up has been proposed as an alternative approach [5, 6, 38]. In our simulations, we demonstrate that this method offers the most robust control of Type I error and yields unbiased estimates under the null hypothesis. This strategy, however, has been criticized because the win‐loss parameters depend on the length of follow‐up, potentially introducing bias in effect size estimates [39]. Our results confirm this concern (Figure 5), but only under the alternative hypothesis. Furthermore, we show that the last common visit approach leads to a loss of power, which can become substantial as dropout rates increase. Multiple imputation of the missing longitudinal data may help recover power, but if assumptions are made about the survival time of those with missing data, this can inflate the Type I error rate. A possible refinement is to impute missing values only for patients known to be alive, in combination with the last common visit approach, or to jointly impute survival time. Moreover, in our study, we used only five imputations due to computational limitations. Increasing the number of imputations is generally advisable because it improves the stability of estimated standard errors, confidence intervals, and *p* values, although it remains unclear whether this would materially affect Type I error rates. More significantly, multiple imputation relies on the assumption that missing data are MAR, whereas missing longitudinal outcomes resulting from death could arise from an MNAR mechanism. Consequently, residual bias and inflated Type I error rates may persist despite careful imputation strategies, while any gains in precision may remain modest.

Our study has several limitations that should be considered. First, all methods were evaluated using a simulation model based on ALS data characterized by limited censoring and missingness. As a result, the absolute trade‐offs between methods may differ under alternative data‐generating mechanisms, in settings with more extensive censoring, or when the strength of the association between the longitudinal and time‐to‐event outcomes differs [36]. Second, our study was restricted to approaches that have been used in previous ALS studies. Other, more recently proposed methods—such as those based on inverse probability of censoring weighting (IPCW) to handle right censoring [40] and its recent extensions to accommodate multiple censored outcomes [41]—may offer potential improvements over the last common visit approach. Finally, the longitudinal outcome in our study was defined as the change from baseline. Alternative definitions have been proposed, however, such as time‐averaged change from baseline or the use of actual observed scores. Evaluating how these different outcome definitions affect generalized pairwise comparisons, particularly with respect to statistical power and efficiency, would be a valuable direction for future research.

In conclusion, we conducted a comprehensive simulation study to evaluate the operating characteristics of various missing data strategies for hierarchical endpoints commonly used in clinical trials. Longitudinal outcomes with repeated assessments play a central role in trials across many therapeutic areas—particularly in neurodegenerative diseases—where generalized pairwise comparisons may help address informative missingness arising from disease progression and differential survival. Our findings demonstrate that certain strategies can introduce substantial bias and inflate Type I error. We provide practical guidance for trialists to select a robust primary analysis strategy and to pre‐specify appropriate sensitivity analyses in their statistical analysis plan.

## Funding

This work was supported by the Dutch Research Council (NWO; VENI 09150162210036) and the US Department of Defense (grant AL230152).

## Conflicts of Interest

The authors declare no conflicts of interest.

## Supporting information


**Data S1:** Supporting Information.

**Data S2:** Supporting Information.
**e‐Table 1.** Parameter estimates used to simulate time‐to‐event and longitudinal data.
**e‐Figure 1.** Empirical power as function of total sample size of the combined assessment of function and survival (CAFS).
**e‐Figure 2a.** Bias under the null hypothesis ($\delta_{0,\text{LTFU}}$ = 10%; $\delta_{2}=1.00$).
**e‐Figure 2b.** Bias under the null hypothesis ($\delta_{0,\text{LTFU}}$ = 0%; $\delta_{2}=0.93$).
**e‐Figure 2c.** Bias under the null hypothesis ($\delta_{0,\text{LTFU}}$ = 0%; $\delta_{2}=1.00$).
**e‐Figure 3a.** Empirical one‐sided Type 1 error ($\delta_{0,\text{LTFU}}$ = 10%; $\delta_{2}=1.00$).
**e‐Figure 3b.** Empirical one‐sided Type 1 error ($\delta_{0,\text{LTFU}}$ = 0%; $\delta_{2}=0.93$).
**e‐Figure 3c.** Empirical one‐sided Type 1 error ($\delta_{0,\text{LTFU}}$ = 0%; $\delta_{2}=1.00$).
**e‐Figure 4.** Bias and empirical statistical power ($\delta_{0,\text{LTFU}}$ = 0%; $\delta_{2}=0.93$).

**Data S3:** Supporting Information.

## Data Availability

The data that supports the findings of this study are available in the Supporting Information of this article.
