# Supplementary material for: Comparing Missing Data Strategies for Generalized Pairwise Comparisons in Randomized Clinical Trials: A Simulation Study
Source: Stat Med. 2026 Sep 17;45(20-22):e70737. doi: 10.1002/sim.70737 (PMC13584238; doi:10.1002/sim.70737)
Supplement: Supplementary file 1 [file SIM-45-0-s001.docx]

**Supplementary material**: *“Comparing Missing Data Strategies for Generalized Pairwise Comparisons in Randomized Clinical Trials: A Simulation Study”*

**TABLE OF CONTENTS	Page**

**e-Table 1**. Simulation parameters based on PRECISION-ALS	2

**e-Figure 1**. Sample size calculation	3

***Alternative scenarios for manuscript Figure 3***

**e-Figure 2a**. Bias under the null hypothesis ($\delta_{0,LTFU}$ = 10%; $\delta_{2}=1.00$)	4

**e-Figure 2b**. Bias under the null hypothesis ($\delta_{0,LTFU}$ = 0%; $\delta_{2}=0.93$)	5

**e-Figure 2c**. Bias under the null hypothesis ($\delta_{0,LTFU}$ = 0%; $\delta_{2}=1.00$)	6

***Alternative scenarios for manuscript Figure 4***

**e-Figure 3a**. Empirical one-sided Type 1 error ($\delta_{0,LTFU}$ = 10%; $\delta_{2}=1.00$)	7

**e-Figure 3b**. Empirical one-sided Type 1 error ($\delta_{0,LTFU}$ = 0%; $\delta_{2}=0.93$)	8

**e-Figure 3c**. Empirical one-sided Type 1 error ($\delta_{0,LTFU}$ = 0%; $\delta_{2}=1.00$)	9

***Alternative scenarios for manuscript Figure 5***

**e-Figure 4**. Bias and empirical statistical power ($\delta_{0,LTFU}$ = 0%; $\delta_{2}=0.93$)	10

**e-Table 1.** Parameter estimates used to simulate time-to-event and longitudinal data

| **Parameter** | **PRECISION-ALS**  (N = 4,927) | ***p*-value** |
| --- | --- | --- |
| *Longitudinal process* |  |  |
| ***Random effects*** |  |  |
| Variance intercept ($\sigma_{1}^{2}$) | 36.76414 | - |
| Variance slope ($\sigma_{2}^{2}$) | 177.17136 | - |
| Variance slope^2^ ($\sigma_{3}^{2}$) | 28.65381 | - |
| Covariance intercept - slope $\sigma_{1}^{2}$ ($\sigma_{1,2}^{2}$) | 28.10447 | - |
| Covariance intercept - slope^2^ ($\sigma_{1,3}^{2}$) | -14.70735 | - |
| Covariance slope - slope^2^ ($\sigma_{2,3}^{2}$) | -58.26383 | - |
| Residual variance ($\sigma_{e}^{2}$) | 5.414891 | - |
| ***Fixed effects*** |  |  |
| Intercept ($\beta_{0}$) | 39.24208 | *p* < 0.001 |
| Slope in years ($\beta_{1}$) | -14.32917 | *p* < 0.001 |
| Slope in years^2^ ($\beta_{2}$) | 1.30610 | *p* < 0.001 |
| *Time-to-event process* |  |  |
| Weibull intercept ($\gamma_{0}$) | 0.2449671 | *p* < 0.001 |
| Weibull shape parameter ($\rho$) | 1.0502200 | *p* = 0.155 |
| Association parameter $m_{i}$($\alpha_{1}$) | -0.0879232 | *p* < 0.001 |
| Association parameter $m_{i}^{'}$($\alpha_{2}$) | -0.0486237 | *p* < 0.001 |

* Table reproduced from “*van Eijk RPA, et al. Natural history of the revised ALS functional rating scale and its association with survival: the PRECISION-ALS Extant Study. Amyotroph Lateral Scler Frontotemporal Degener 2025;26:30-40.*”

**e-Figure 1**. Empirical power as function of total sample size of the combined assessment of function and survival (CAFS).

**e-Figure 1 legend**. The empirical power for different sample sizes was estimated by simulating a 1:1 randomized trial in which active treatment results in a 25% reduction in disease progression rate with an additional 25% reduction in death rate over a 12-month period. Each sample size was simulated 15,000 times; empirical power reflects how many times a *p* < 0.05 was observed. The error bars depict the simulation uncertainty; the solid line is the meta-analyzed association between total sample size and empirical power together with its 95% confidence interval. A total sample size of 318 was deemed sufficient to detect the hypothesized treatment effect with at least 80% power.

**e-Figure 2a**. Bias under the null hypothesis ($\delta_{0,LTFU}$ = 10%; $\delta_{2}=1.00$)

**e-Figure 2b**. Bias under the null hypothesis ($\delta_{0,LTFU}$ = 0%; $\delta_{2}=0.93$)

**e-Figure 2c**. Bias under the null hypothesis ($\delta_{0,LTFU}$ = 0%; $\delta_{2}=1.00$)

**e-Figure 3a**. Empirical one-sided Type 1 error ($\delta_{0,LTFU}$ = 10%; $\delta_{2}=1.00$)

**e-Figure 3b**. Empirical one-sided Type 1 error ($\delta_{0,LTFU}$ = 0%; $\delta_{2}=0.93$)

**e-Figure 3c**. Empirical one-sided Type 1 error ($\delta_{0,LTFU}$ = 0%; $\delta_{2}=1.00$)

**e-Figure 4**. Bias and empirical statistical power ($\delta_{0,LTFU}$ = 0%; $\delta_{2}=0.93$)
